# Prevalence and risk factors of burnout among Lebanese community pharmacists in the era of COVID-19 pandemic: results from the first national cross-sectional survey

**DOI:** 10.1186/s40545-021-00393-w

**Published:** 2021-12-24

**Authors:** Dalal Youssef, Janet Youssef, Hamad Hassan, Linda Abou-Abbas

**Affiliations:** 1 Preventive Medicine Department, Ministry of Public Health, Beirut, Lebanon; 2 Clinical Trial Program, Ministry of Public Health, Beirut, Lebanon; 3 Institut de santé publique d’épidémiologie et de développement (ISPED), Bordeaux University, Bordeaux, France; 4 Present Address: Al Zahraa Hospital University Medical Center, Beirut, Lebanon; 5 Present Address: Ministry of Public Health, Beirut, Lebanon; 6 Faculty of Medical Sciences, Neuroscience Research Center, Lebanese University, Beirut, Lebanon; 7 Present Address: Epidemiological Surveillance Program, Ministry of Public Health, Beirut, Lebanon

**Keywords:** Burnout, Community pharmacists, COVID-19, Prevalence, Risk factors, Lebanon

## Abstract

**Background:**

Community pharmacists (CPs) are increasingly facing occupational challenges as a result of the COVID-19 pandemic, putting them at high risk of burnout. This study aimed to assess the prevalence of burnout among Lebanese CPs during the COVID-19 pandemic and to identify its associated factors.

**Methods:**

An online survey was conducted among Lebanese CPs between February 1st and March 30, 2021. Collected data included information on sociodemographic characteristics, exposure and work-related variables, the Copenhagen burnout inventory (CBI), and the COVID-19 threat perception scale. Prevalence of burnout was calculated. Multiple logistic regressions were performed to identify the factors associated with the three burnout domains.

**Results:**

A total of 387 CPs participated in the survey. Of the total, 53.7% were females; and 43.2% were aged less than 40 years old. The prevalence of moderate-to-high personal, work-related and client-related burnout was 77.8%, 76.8, and 89.7%, respectively. Younger age, staff pharmacist, working more than 40 h per week, high perceived COVID-19 threat were associated with a moderate-to-high likelihood of burnout in all three domains. However, altruistically accepting the risks of caring for COVID-19 patients was the only variable that was associated with a lower likelihood of burnout in all three domains.

**Conclusion:**

An alarming prevalence of personal, work-related and client-related burnout was revealed among Lebanese CPs. This study has many implications for practice and provides a framework for establishing policy interventions to reduce burnout levels among Lebanese CPs. Preventive strategies and interventions on individual and organizational basis are recommended.

## Background

Burnout syndrome is affecting all healthcare workforce disciplines and community-based pharmacists (CPs), representing the third largest health care professional group outnumbered only by physicians and nurses, are no exception [1–4]. Over the last three decades, the role of the community pharmacist (CPs) has evolved from distributing drugs to improving patient-centered care and pharmacist–patient engagement [5]. While the types of patient care services provided by CPs vary greatly by country, an increasing number of pharmacists are providing critical emergency medication refills, prescription renewals/extensions, dose or formulation changes, therapeutic substitution, prescribing for minor ailments, initiation of prescription drug therapy, ordering and interpreting laboratory tests, immunization and drug administration. However, the transition from product-based to service-based care was confronted by several challenges experienced through interacting with the workplace environment [6]. These challenges were described as inducing psychosocial stress and burnout [7–10]. Of note, stressors confronted by CPs mirror those of physicians, including an ineffective work environment, the burden of non-clinical and administrative duties, and excessive workloads combined with lack of resources [11]. The negative consequences of CPs burnout could affect patient care [12] and can lead to CPs poor self-care, substance abuse, depression, and suicidal ideation [8, 13–15].

In the context of CPs population, burnout is not deeply explored. A systematic review appraised that the prevalence of overall burnout among pharmacists ranged from 52 to 61% [16]. Another study conducted before the Coronavirus disease (COVID-19) pandemic with the assistance of the American Pharmacists Association (APhA) has estimated that 75% of CPS suffered from burnout [17]. Given the large heterogeneity in the role and responsibilities of CPs within health systems and across practice settings [18], it is essential to take into consideration differences among pharmacy practice settings that could contribute to differing levels of burnout.

Since the onset of COVID-19, increased demand for medical services has strained worldwide the health care systems to their limits [19]. Subsequently, the majority of non-urgent operations and medical services in health facilities were canceled or suspended to allow the healthcare system to manage the soar of severe cases of COVID-19. As result, the public turned to CPs since they remain the most accessible face-to-face primary healthcare provider [20]. Some countries have recognized the pharmacy as an ideal access point for patients and allowed CPs to run COVID-19 testing along with COVID-19 vaccination services. These inflicted duties on the shoulder of CPs have increased their risk of burnout. Several studies have described the psychological impacts of the COVID-19 pandemic on healthcare workers [21], but CPs were rarely included in such studies.

Lebanon, a small country of the Middle East, has one of the world's highest pharmacist-to-population ratios (20.3/10,000 people) [22]. Nonetheless, workforce evaluations revealed major problems about Lebanese pharmacist distribution, practice settings, and regulation. Since CPs sites are privately owned, they are not evenly distributed throughout Lebanese provinces. As per the Lebanese law of 1950, pharmacist registration within the Order of Pharmacists (OPL) is a requirement for practicing the profession of pharmacy. In addition, the payment of an annual fee and the enrollment in the compulsory continuing education program are requisite to preserve their registration. CPs practice is also supervised by inspectors from the OPL in collaboration with inspectors from the Ministry of Public Health (MOPH). Under the Lebanese OPL and the MOPH laws that regulate pharmacy practice, CPs are requested to be present all the time during the pharmacy's working hours. Of note, a technician can dispense a medicine to a patient without consulting the on-duty CPs and Lebanese patients can buy any non-controlled medication despite its classification as a prescription medication in other countries. In addition, CPs have other responsibilities such as storing and supplying adequate stocks of medicines, as well as counseling, educating the public, and promoting disease prevention and infection control. A pharmacy may only be set up with the permission of the Minister of Public Health. The establishment of a community pharmacy should be licensed by the MOPH and this permission is granted only to a Lebanese pharmacist who holds a license to practice pharmacy profession in Lebanon. A non-Lebanese pharmacist from other nationalities is required to meet the same conditions set out for Lebanese pharmacist as well as to be associated to a country that treats Lebanese pharmacists likewise based on an agreement of reciprocity between the two countries. A CP should also be certified for at least 10 years. Of note, the pharmacy certificate obtained outside Lebanon is not approved by the MOPH committee of equivalence unless the study program of the university that awarded it is equivalent to that of the Lebanese institutions [23].

Over the last two years, Lebanon’s economic collapse crisis coupled with political crisis and the COVID-19 pandemic, have led to dreadful consequences on the pharmacy profession [24]. Under such circumstances, it comes as no surprise the closure of more than 200 pharmacies, the shortage in the supply of essential pharmaceutical products and the risk of shut down of additional 1000 pharmacies. It is also worth noting that there has been an upsurge in the occurrence of pharmacy theft, adding to the sufferings of Lebanese CPs. Besides, the economic collapse and the Lebanese currency value steep loss combined with the medicines inflation prices and the imposed lockdown escalate the Lebanese population concerns towards an imminent medicines shortage in the Lebanese market. Turned into panic mode, the Lebanese population experienced an unprecedented pace to purchase medicines. These stressors such as the soaring demand for medicines, limited supply chain, the financial crisis, the threat of COVID-19, and the increased responsibilities created the level of burnout among CPS. Furthermore, precautions measures implemented in the pharmacy setting, managing crowding, and social distancing have been also shown to have the potential to increase work-related stress [25, 26]. With healthcare workers reporting psychological impacts from the COVID-19 pandemic, burnout syndrome has not been assessed among Lebanese CPs. Therefore, it is of great interest to assess the extent of burnout among CPs and to identify its associated factors.

## Methods

### Study design and population

A national web-based cross-sectional study was conducted among Lebanese CPs over a period of 2 months extending from February 1st till the end of March 2021. Participants were identified via the list of registered CPs provided by the OPL and were electronically invited to participate. Before their enrollment in the study, CPs were contacted via phone call and notified about the survey and its purpose. Upon their agreement to participate, an online questionnaire using a Google form was sent to them via emails or WhatsApp. They were also invited to share with their colleagues the survey link that included a brief explanation of the study purpose and the electronic informed consent.

All CPs of either gender or profile (owner, manager, or staff pharmacist) working in pharmacy setting at the time of the survey, who had access to the internet and who agreed to participate in the study were eligible for participation. These professionals were defined as the pharmacy team. Exclusion criteria were defined as follows: clinical pharmacists, retired CPs, those who were out of the country at the time of the survey, trainees and pharmacy students or other professionals (e.g., dietician, beautician), as well as those who are not currently practicing. Pharmacists who refused to give informed consent were also excluded from the study. There was no age limitation.

All methods were performed following the relevant guidelines and regulations such as the STROBE (Strengthening the Reporting of Observational Studies in Epidemiology) guidelines for reporting observational studies [27]. Since the study has no foreseeable risks, written consent was obtained in an electronic format. Participants have not received any compensation for their participation in the study.

### Ethical consideration

Participation in this survey was voluntary and participants were allowed to withdraw from the study at any time. Electronic informed consent was obtained for each participant. All information was gathered anonymously and handled confidentially. The study design assured adequate protection of study participants. None of the survey questions asked for information that could harm the participant in any way.

### Sample size calculation

The digital Raosoft sample size calculator was used to calculate the sample size of the study, based on a total population size of 4185 community pharmacies registered with OPL, a 95% confidence level and an absolute error of 5%, a minimal sample of 352 pharmacists was required.

### Instrumentation

A 58-item questionnaire was developed by the study authors and was reviewed by a panel of experts who were asked to evaluate its content validity based on the relevance, coverage, and representativeness of the items. Of the total items, three were rated irrelevant, thus, they were omitted from the questionnaire. Then, the questionnaire was translated to the Arabic language by a bilingual Arabic–English translator whose first language was Arabic. The translator was asked to develop an Arabic version of the questionnaire that can be understood by all Arabic-speaking individuals. A pre-final version of the translation was drafted and was administered as a pilot study to 20 CPs to evaluate the comprehensibility of the questionnaire. After receiving their feedback, minor linguistic edits were made.

The survey consisted of three sections: (a) sociodemographic characteristics; (b) exposure to severe acute respiratory syndrome coronavirus 2 (SARS-COV-2); (c) COVID-19 threat perception scale, and (d) Copenhagen Burnout scale (A-CBI).

- Sociodemographic characteristics: gender, age, marital status, profile, residency, education level, health status, history of medical illnesses, health status of people living with the participant, and the presence of an elderly or dependent child at home.
- Exposure to SARS-COV-2. CPs were asked to answer on a yes or no basis whether they were frontline workers in COVID-19, have been tested for COVID-9, previously diagnosed as confirmed COVID-19 case, and had a family member or colleague infected by SARS-COV-2.
- COVID-19 perceived threat: this tool was developed by Chong et al. to assess the COVID-19 risk perception among HCWs [28]. It consisted of 10 items where nine of these items described HCWs' perception toward COVID-19 threat and one item related to altruistic acceptance of COVID-19 risk. Ratings were given based on a five-point Likert scale (1 = strongly disagree, 2 = disagree, 3 = neutral, 4 = agree, 5 = strongly angry). Responses were dichotomized into positive responses ‘agree’ or ‘strongly agree’, while ‘strongly disagree’, ‘disagree’, and ‘not sure’ were considered negative. In our study, the Cronbach’s alpha of this scale was equal to 0.703.
- The Arabic version of Copenhagen Burnout scale A-CBI: the cross culturally adapted 19-item CBI scales by Youssef et al. was used in the current study to evaluate the three aspects of burnout: personal-related (6 items), work-related (7 items), and client-related (6 items) burnout [29]. Twelve items have responses categories according to frequency ranging as follows: (0 = never/almost, 25 = seldom, 50 = sometimes, 75 = often, 100 = always). Seven questions have answers have responses of intensity ranging from ‘a very low degree’ to ‘to a very high degree’. Scores of less than 50 are considered ‘no/low’, 50 to 74 are considered ‘moderate’, 75–99 are high, and a score of 100 is considered ‘severe’ burnout. Moreover, score were dichotomized as follows: a score ˂50 is considered no/low burnout level whereas a score ≥ 50 is considered moderate/severe burnout level. [30]. In the current study, Cronbach’s alpha reliability coefficients of the CBI subscales were high (personal burnout α = 0.91; work-related burnout α = 0.85; and client-related burnout α = 0.89).

### Statistical analysis

The generated data on an excel spreadsheet were transferred to the statistical software IBM SPSS® software (Statistical Package for Social Sciences) version 24.0 for analysis. Before analyzing it, the database was weighed according to the governorate where pharmacy is located, based on the list provided by the OPL. Descriptive statistics were reported using frequency with percentages for categorical variables and mean along with standard deviation for continuous variables. For the bivariate analysis of continuous variables, the Chi-2 test was used to compare categorical variables. All variables that showed a *p*-value < 0.2 in the bivariate analysis were included in the multivariable analysis as independent variables. Four logistic regressions using were conducted to identify the correlates of each of the CBI scales using overall burnout, personal burnout, work-related burnout and client-related burnout, respectively, as dependent variable. A *p*-value less than 0.05 is considered statistically significant.

## Results

### Baseline information of the participants

A total of 387 CPs participated in the survey. Of the total, 53.7% were females; 60.5% were married, 43.2% were aged less than 40 years old, and 65.9% were residents of urban areas. Around half of the participants hold a BS degree in pharmaceutical sciences, had less than 10 years of professional experience (56.9%), working more than 40 h per week (59.9%), having a monthly income more than 2 million Lebanese pounds (53.9%) and were pharmacy’ owner (55.3%). Most of surveyed CPs worked in pharmacies located in Mount-Lebanon governorate, which is mainly operating around 50–120 h per week (81.6%). Of note, 77.5% have a good health status. In terms of family members, nearly half of participants had currently a dependent child (55.5%) or were living with the elderly (51.4%) or a family member with comorbidities living with them at home (59.4%). In terms of exposure, 76.7% of them were tested for COVID-19 and 23% were diagnosed with COVID-19. A detailed description of the baseline characteristics of the surveyed CPs is presented in Table 1.

**Table 1:** Sociodemographic characteristics of surveyed Lebanese community pharmacists (*N* = 387)

	*n*	%
Gender		
Male	179	46.30%
Female	208	53.70%
Age (years)		
Less than 40 y	254	65.60%
≥ 40 y	133	34.40%
Marital status		
Unmarried*	153	39.50%
Married	234	60.50%
Pharmacy location		
North and Akkar	48	12.40%
Mount Lebanon	145	37.50%
Beirut	60	15.50%
South and Nabatyeh	79	20.40%
Bekaa and Baalbeck-Hermel	55	14.20%
Urbanicity (residency)		
Rural	132	34.10%
Urban	255	65.90%
Years of experience		
0–10 years	220	56.90%
More than 10 years	167	43.20%
Profile		
Staff pharmacist	135	34.90%
Owner	214	55.30%
Manager	38	9.80%
Highest education level		
BS pharmacy	216	55.80%
Other (Master, PharmD, PhD…)	171	44.20%
Number of hours per week pharmacy is open		
Less than 50 h	40	10.40%
50–120 h	316	81.60%
7 days 24/24 h	31	8.00%
Pharmacist working hours		
Less than 40 h		
40 h or more	155	40.10%
Household income	232	59.90%
< 2 millions	178	46.10%
> 2 millions	209	53.9%
Health status		
Fair and below	87	22.50%
Good and above	300	77.50%
Presence of dependent child		
No	172	44.50%
Yes	215	55.50%
Presence of elderly people at home		
No	188	48.60%
Yes	199	51.40%
Living with family member with comorbidities		
No	157	40.60%
Yes	230	59.40%
Ever tested for COVID-19		
No	90	23.30%
Yes	297	76.70%
Personal history of COVID-19 diagnosis		
No	298	77.00%
Yes	89	23.00%
Family member/friend ever diagnosed with COVID-19		
No	256	66.10%
Yes	131	33.90%
Colleague ever diagnosed with COVID-19		
No	35	9.00%
Yes	352	91.00%

*N* frequency, % percentage, *Other included divorced or widowed

### Description of the scales

The means and standard deviations of personal burnout work-related burnout and client-related burnout scales were 67.17 (SD = 16.82), 67.02 (SD = 14.15), and 69.38 (SD = 20.78), respectively. A detailed description of the scales items is presented in Table 2.

**Table 2:** Descriptive statistics of the scales used in the study

#	Scale items	Mean	S.D
TPS	Threat perception scale	**36.68**	**1.92**
Threat1	My job puts me at great risk	3.96	0.63
Threat2	I feel more stress at work	3.86	0.77
Threat3	I have little control over whether I get infected or not	3.01	0.10
Threat4	I have little chance of survival if I were to get SARS	2.16	0.48
Threat5	I think of resigning because of SARS	2.16	0.48
Threat6	I am afraid that I will pass SARS to others	4.23	0.83
Threat7	My family and friends are worried they get infected through me	3.85	0.53
Threat8	People avoid my family because of my work	4.03	0.93
Threat9	I am afraid of falling ill with SARS-COV-2	3.82	0.72
ALtru1	I accept the risk of caring for a SARS-COV-2 patient	3.63	0.74
CBI	Copenhagen Burnout Inventory Scale	**65.34**	**17.39**
	Personal burnout	**67.17**	**16.82**
PB1	How often do you feel tired?	66.41	16.56
PB2	How often you are physically exhausted?	66.41	16.56
PB3	How often you are emotionally exhausted?	66.41	16.56
PB4	How often do you think: "I can’t take it anymore"?	69.06	12.48
PB5	How often do you feel worn out?	67.38	16.88
PB6	How often do you feel weak and susceptible to illness?	67.38	16.88
	Work-related burnout	**67.02**	**14.15**
WB1	Is your work emotionally exhausting?	64.08	13.42
WB2	Do you feel burnt out because of your work?	68.67	11.18
8WB3	Does your work frustrate you?	69.12	11.91
WB4	Do you feel worn out at the end of the working day?	69.77	11.53
WB5	Are you exhausted in the morning at the thought of another day at work?	66.15	12.24
WB6	Do you feel that every working hour is tiring for you?	69.51	11.26
WB7	Do you have enough energy for family and friends during leisure time? ^R^	66.60	12.09
	Client burnout	**69.38**	**20.78**
CB1	Do you find it hard to work with clients?	68.27	21.39
CB2	Do you find it frustrating to work with clients?	62.82	22.39
CB3	Does it drain your energy to work with clients?	67.67	23.53
CB4	Do you feel that you give more than you get back when you work with clients?	68.82	24.29
CB5	Are you tired of working with clients?	72.03	24.98
CB6	Do you sometimes wonder how long you will be able to continue working with clients?	69.30	21.23

M:mean, SD: standard deviation, ^**R**^: reversed coding

### Risk perceptions and altruistic acceptance of risk during the COVID-19 pandemic

**Fig. 1:**
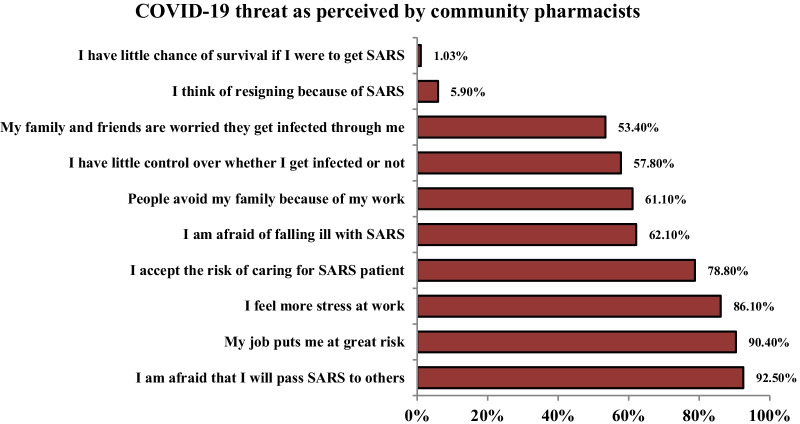
Community pharmacists’ perception of COVID-19 threat (N = 387)

More than 90% of surveyed CPs believed that their job was putting them at risk and were afraid to transmit the COVID-19 to their families and friends. In addition, 86.1% of them felt extra stress at work and 62.1% were afraid of falling ill with COVID-19, while 61.1% were worried that people avoid their families because of their work. More than half of CPs considered they had little control over being infected or not. 53.4% of participants were concerned that their families and friends feared to get infection through them. Only 1.03% of CPs believed that they would die if they get infected and 4.9% thought about resigning because of COVID-19. Most participants (78.8%) altruistically accepted taking the risk of caring for COVID-19 patients (Fig. 1).

### Prevalence of burnout among Lebanese community pharmacists

**Fig. 2:**
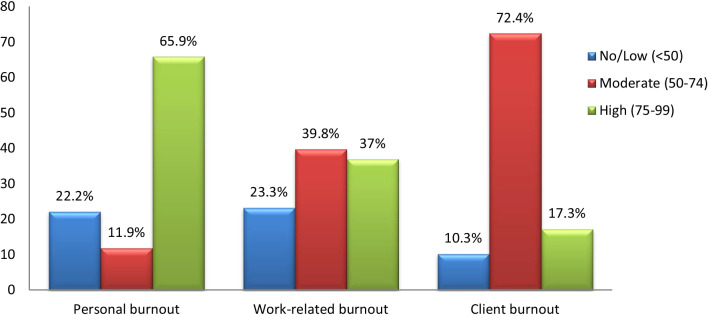
Prevalence of burnout among Lebanese community pharmacists (N = 387)

The prevalence of CPs reporting high personal burnout level was 65.9% while 37% and 17.3% of CPs reported high burnout level on workload burnout and client-burnout subscales, respectively. The overall prevalence of CPs reporting moderate or higher burnout level was 89.7% for the client-burnout subscale, followed by personal burnout (77.8%), and work-related burnout (76.8%) (Fig. 2).

### Factors associated with personal burnout

Younger age (aOR = 1.792, 95% CI (1.342–1.904)), female gender (aOR = 2.632, 95% CI (1.913–4.187)), staff pharmacist (aOR = 2.116, 95% CI (1.618–3.807)), working more than 40 h per week (aOR = 1.663, 95% CI (1.321–2.732)), having a dependent child (aOR = 2.632, 95% CI (1.913–4.187)), insufficient sleeping hours (aOR = 3.219, 95% CI (2.013–6.127)), having a colleague diagnosed with COVID-19 (aOR = 1.852, 95% CI (1.347–2.786)) and high perceived COVID-19 threat (aOR = 1.852, 95% CI (1.347–2.786)) were more likely to have moderate-to-high personal burnout level compared to their counterparts. However, being married (aOR = 0.876, 95% CI (0.669 0.942)), having high socioeconomic status (aOR = 0.367, 95% CI (0.218–0.605)), and altruistically accepting the risks of caring for COVID-19 patients (aOR = 0.812, 95% CI (0.623–0.918)) were significantly associated with a lower likelihood of personal burnout level (Table 3).

**Table 3:** Factors associated with personal burnout scale among CPs

	No/low	Moderate to high				Confidence interval 95%	
	*n* (%)	*n* (%)	Total	*p*-value	aOR	Lower	Upper
Gender				**0.022**			
Male	48 (26.8%)	138 (73.2%)	179 (46.3%)		1.00		
Female	38 (18.3%)	170 (81.7%)	208 (53.7%)		2.632	1.913	4.187
Age (years)				**0.023**			
≥ 40 y	50 (37.5%)	83 (62.5%)	133 (34.4%)		1.00		
Less than 40 y	36 (14.2%)	218 (85.8%)	254 (65.6%)		1.792	1.342	1.904
Marital status				**0.048**			
Single and other^a^	39 (25.4%)	114 (74.6%)	153 (39.5%)		1.00		
Married/engaged	47 (20%)	187 (80%)	234 (60.5%)		0.876	0.669	0.942
Profile				**0.039**			
Owner	54 (25.2%)	160 (74.8%)	214 (55.3%)		1.00		
Manager	8 (21.1%)	30 (79.9%)	38 (9.8%)		1.213	0.907	1.813
Staff pharmacist	24 (17.7%)	111 (82.3%)	135 (34.9%)		2.116	1.618	3.807
Pharmacist working hours				**0.026**			
Less than 40 h	32 (20.6%)	123 (79.4%)	155 (40.1%)		1.00		
40 h or more	54 (23.2%)	178 (76.8%)	232 (59.9%)		1.663	1.321	2.732
Subjective classification of the current economic status				** < 0.001**			
Low	49 (16.9%)	242 (87.1%)	278 (46.4%)		1.00		
Middle	16 (21.3%	63 (78.7%)	80 (20.7%)		0.871	0.689	0.914
High	21 (72%)	187 (89.9%)	29 (43.6%)		0.367	0.218	0.605
Presence of dependent child				** < 0.001**			
No	66 (38.9%)	106 (61.1%)	172 (44.5%)		1.00		
Yes	20 (9.3%)	195 (90.7%)	215 (55.5%)		4.017	3.818	7.432
Personal history of COVID-19 diagnosis				0.079			
No	70 (23.4%)	228 (76.6%)	298 (77%)				
Yes	16 (17.9%)	73 (82.1%)	89 (23%)				
Family member/friend ever diagnosed with COVID-19				0.102			
No	55 (21.5%)	201 (79.5%)	256 (66.1%)				
Yes	31 (23.7%)	100 (76.3%)	131 (33.9%)				
Colleague ever diagnosed with COVID-19				**0.006**			
No	19 (54.3%)	16 (45.7%)	35 (9%)		1.00		
Yes	67 (19.1%)	285 (80.9%)	352 (91%)		4.73	2.782	7.112
Sleeping hours				** < 0.001**			
≥ 6 h	54 (24.5%)	166 (75.5%)	220 (56.8%)		1.00		
< 6 h	32 (19.1%)	135 (86.2%)	167 (43.2%)		3.219	2.013	6.127
Altruism				**0.042**			
Disagree	20 (24.4%)	62 (75.6%)	82 (21.25%)		1.00		
Agree	66 (21.6%)	239 (78.4%)	305 (78.8%)		0.812	0.623	0.918
Threat perception scale				**0.032**	1.852	1.347	2.786

*n* frequency, % percentage, ^a^Other included divorced or widowed. C.I: confidence interval, aOR: adjusted odds ratio

### Factors associated with work-related burnout

Younger age CPs (aOR = 2.132, 95% CI (1.168–3.005)), staff pharmacist (aOR = 4.12, 95% CI (2.192–6.117)), having extensive working hours (aOR = 1.709, 95% CI (1.221–3.405)), having a dependent child (aOR = 2.361, 95% CI (1.765–3.812)), dealing with COVID-19 cases (aOR = 1.912, 95% CI (1.682–3.829)), working in a pharmacy which is operating more than 50 h per week (aOR = 4.178, 95% CI (2.781–6.553)), having insufficient sleeping hours (aOR = 2.918, 95% CI (1.812–5.218)), having a colleague diagnosed with COVID-19 (aOR = 3.819, 95% CI (2.011–7.415)) and high perception of COVID-19 threat (aOR = 2.853, 95% CI (1.473–3.885) were more likely to have moderate-to-high work-related burnout. However, high socioeconomic status (aOR = 0.367, 95% CI (0.218–0.605)), more than 10 years of experience (aOR = 0.761, 95% CI (0.532–0.898)), altruistically accepting the risks of caring for COVID-19 patients (aOR = 0.722, 95% CI (0.512–0.909)) were significantly associated with a lower likelihood of work-related burnout compared to their counterparts (Table 4).

**Table 4:** Factors associated with work-related burnout scale among CPs

	No/low	Moderate/high				95% Confidence interval	
	*n* (%)	*n* (%)	Total	*p*-value	aOR	Lower	Upper
Age (years)				**0.023**			
≥ 40 y	42 (31.6%)	91 (68.4%)	133 (34.4%)		1.00		
Less than 40 y	48 (18.9%)	206 (91.1%)	254 (65.6%)		2.132	1.168	3.005
Years of experience				**0.013**			
0–10 years	39 (17.7%)	181 (82.3%)	220 (56.9%)		1.00		
More than 10 years	51 (30.5%)	116 (69.5%)	167 (43.2%)		0.761	0.532	0.898
Profile				**0.011**			
Owner	65 (30.3%)	149 (69.7%)	214 (55.3%)		1.00		
Manager	14 (36.8%)	24 (63.2%)	38 (9.8%)		1.812	0.923	3.107
Staff pharmacist	11 (8.2%)	124 (91.8%)	135 (34.9%)		4.12	2.192	6.117
Number of hours per week pharmacy is open				** < 0.001**			
Less than 50 h	20 (50%)	20 (50%)	40 (10.4%)		1.00		
50–120 h	65 (20.5%)	251 (79.5%)	316 (81.6%)		3.128	2.129	5.338
7 days 24/24 h	5 (16.1%)	26 (83.9%)	31 (8%)		4.178	2.781	6.553
Pharmacist working hours				** < 0.001**			
Less than 40 h	58 (37.4%)	97 (62.6%)	155 (40.1%)		1.00		
40 h or more	32 (13.7%)	200 (86.3%)	232 (59.9%)		1.709	1.221	3.405
Presence of underlying condition				** < 0.001**			
No	73 (24.4%)	225 (75.6%)	298 (77.00%)		1.00		
Yes	17 (19.1%)	72 (80.9%)	89 (23%)		1.821	1.239	3.011
Presence of dependent child				** < 0.001**			
No	70 (40.7%)	102 (59.3%)	172 (44.5%)		1.00		
Yes	20 (9.3%)	195 (90.7%)	215 (55.5%)		2.361	1.765	3.812
Dealing with COVID-19 case				**0.032**			
No	60 (29.7%)	142 (70.3%)	202 (48.1%)		1.00		
Yes	30 (16.3%)	155 (83.7%)	185 (51.9%)		1.912	1.682	3.829
Sleeping hours				** < 0.001**			
≥ 6 h	64 (29.1%)	156 (70.9%)	220 (56.8%)		1.00		
< 6 h	26 (15.5%)	141 (84.4%)	167 (43.2%)		2.918	1.812	5.218
Subjective classification of the current economic status				** < 0.001**			
Low	50 (17.9%)	228 (82.1%)	278 (46.4%)		1.00		
Middle	22 (27.5%)	58 (72.5%)	80 (20.7%)		0.871	0.689	1.914
High	18 (62.1%)	11 (37.9%)	29 (43.6%)		0.367	0.218	0.605
Altruism				**0.042**			
Disagree	66 (80.4%)	16 (19.6%)	82 (21.25%)		1.00		
Agree	24 (7.9%)	281 (92.1%)	305 (78.8%)		0.722	0.512	0.909
Threat perception scale				**0.016**	2.853	1.472	3.885
Colleague ever diagnosed with COVID-19				**0.001**			
No	20 (57.1%)	15 (42.9%)	35 (9%)		1.00		
Yes	70 (19.9%)	282 (80.1%)	352 (91%)		3.819	2.011	7.415

*n* frequency, % percentage, *Other included divorced or widowed

### Factors associated with client-related burnout

Younger age (aOR = 1.792, 95% CI (1.342–1.904)), staff pharmacists (aOR = 3.021, 95% CI (1.892–5.327)), working more than 40 h per week (aOR = 4.302, 95% CI (2.918–7.503)), taking care of COVID-19 cases (aOR = 3.781, 95% CI (1.461–7.412)) and high COVID-19 threat perception (aOR = 2.032, 95% CI (1.283–4.066)) were significantly associated with moderate-to-high client-related burnout level. However, altruistically accepting the risks of caring for COVID-19 patients (aOR = 0.582, 95% CI (0.381–0.765)) was significantly associated with a lower likelihood of client-related burnout level (Table 5).

**Table 5:** Factors associated with the client-related burnout scale

	No/low	Moderate/high				Confidence interval	
	*n* (%)	*n* (%)	Total	*p*-value	aOR	Lower	Upper
Age (years)				**0.023**			
≥ 40 y	31 (23.3%)	102 (76.7%)	133 (34.4%)		1.00		
Less than 40 y	39 (15.4%)	293 (84.6%)	254 (65.6%)		1.792	1.342	1.904
Profile				**0.021**			
Owner	53 (24.7%)	161 (75.3%)	214 (55.3%)		1.00		
Manager	7 (18.4%)	31 (81.6%)	38 (9.8%)		1.322	0.879	3.512
Staff pharmacist	10 (7.4%)	123 (92.6%)	135 (34.9%)		3.021	1.892	5.327
Pharmacist working hours				** < 0.001**			
Less than 40 h	49 (31.6%)	106 (68.4%)	155 (40.1%)		1.00		
40 h or more	21 (9.1%)	211 (89.9%)	232 (59.9%)		4.302	2.918	7.503
Taking care of COVID-19 cases				**0.012**			
No	65 (32.2%)	137 (67.8%)	202 (48.1%)		1.00		
Yes	5 (2.7%)	180 (97.3%)	185 (51.9%)		3.781	1.467	7.412
Altruism							
Disagree	8 (9.5%)	58 (91.5%)	82 (21.25%)		1.00		
Agree	62 (20.3%)	243 (79.7%)	305 (78.8%)		0.582	0.381	0.765
Threat perception				**0.043**	2.032	1.283	4.066

## Discussion

To the best of our knowledge, this study is the first nationwide survey assessing the prevalence of burnout among CPs during the COVID-19 pandemic and examining its associated factors. The prevalence of moderate-to-high personal, work-related and client-related burnout was 77.8%, 76.8, and 89.7%, respectively. Younger age, staff pharmacist, working more than 40 h per week, high perceived COVID-19 threat were associated with a moderate-to-high likelihood of burnout in all three domains. However, altruistically accepting the risks of caring for COVID-19 patients was the only variable that was associated with a lower likelihood of burnout in all three domains.

Our findings showed that the majority of Lebanese CPs suffered from burnout in all the three domains. This was predictable since Lebanese CPs were stranded under a perfect storm that could instigate burnout. The alarming level of burnout among CPs in all three domains stressed the importance of urgent action to tackle such epidemic. Due to the uncertainty of the length of the current pandemic and the ongoing economic crisis, no one can neglect the considerable lasting impact of this syndrome. Our findings were higher than those reported in prior studies. A study conducted among CPs showed that 74.9% of respondents experienced burnout in at least 1 of the 3 subscales of the MBI-HSS [31]. A study conducted in Serbia reported that 44.4% of CPshad high levels of burnout [32]. A systematic review found the prevalence of burnout in pharmacists ranged from 19 to 37% [16]. Another study conducted among pharmacists in Saudi Arabid showed that 25.16% of pharmacists had high emotional exhaustion, 55.97% had high depersonalization, and 63.52% had low scores for personal accomplishment [33]. However, such comparison should be cautious since different scales were used.

Younger age was found to be associated with higher level of burnout in all three domains. This was consistent with the findings of a study conducted in United States by Jones et al. among hospital clinical pharmacy practitioners [8]. This could be explained by the fact that younger pharmacists have the most workload, as the first few years of a pharmacist's career can be filled with night shifts which can contribute to a feeling of burnout. Regarding the role of gender on the development of burnout among CPs, our results showed that female CPs were more likely to experience high level of personal burnout compared to males. Our results were consistent with the findings of a nationwide survey performed among the American Pharmaceutical Association membership which revealed higher level of burnout in women compared to men pharmacists [34].

Compared to pharmacy owners, staff pharmacists had higher odds of burnout in all three domains. These results were in line with the findings of an Italian study which revealed that CPs most exposed to exhaustion were those who played the role of employee compared to those who held the role of holder, manager or other management roles [35]. Such finding was expected, as the staff pharmacists experience a relevant workload and were more exposed to clients than managers and owners, especially in larger pharmacies [36].

With regard to marital status, married CPs were found less likely to suffer from higher burnout compared to unmarried CPs. Consistent with other studies, unmarried respondents had significantly higher exhaustion than married [37]. This could be explained by the fact that CPs receiving support from their partners experience less burnout when compared to those who do not. Of note, one of the suggested ideas found in the literature to overcome burnout was the building of support systems through family to enhance self-esteem and reduce burnout.

With regard to sleeping hours, our study showed that sleeping more than 6 h daily was associated with lower level of personal and work-related burnout. Of note, the role of extensive working hours and sleep deprivation, was reported as risk factor for burnout among CPs in several studies especially when working in pharmacies operating round the clock with night shift [8, 38]. With the economic collapse and the steep loss of the value of the Lebanese currency combined with the inflation of the drug prices, an unprecedented race to purchase medicines from pharmacies was reported. Hence, Lebanese CPs are facing intense workload, extensive working hours, which eventually impact physicians sleeping hours and increase burnout levels [39, 40]. A study conducted prior to the pandemic showed that 33% of the HCWs were screened positive for the sleeping disorder and this was associated with fourfold bigger odds of burnout [41]. These results were in line with the findings among Saudi Arabian pharmacists where sleeping hours per day were significantly correlated with burnout. Of note, sleep disturbances was positively associated with impaired performance, emotional changes, tiredness, loss of concentration, and mood disorders as anxiety or depression [42]. In addition to all of the above, high socioeconomic status (aOR = 0.452, 95% CI (0.238–0.611)) was associated with lower personal and work-related burnout level. Several studies showed a correlation between income satisfaction and burnout. Other studies showed that low annual salary was associated with higher burnout level [37]. In terms of living conditions, having a dependent child was associated with higher personal and work-related burnout level. This could be due to the fears expressed by CPs to transfer the infection to their families. Similarly higher perception of COVID-19 threat, in terms of catching the infection, transmission of the COVID-19, stigmatization of family was associated with higher level of burnout. These findings were not surprising as CPs believed that their job was putting them at risk and were afraid to transmit the infection to others including their families.

Several limitations should be acknowledged in our study. First, the cross-sectional design of our study does not allow us to infer causality or temporal relationship. Secondly, selection bias is possible due to the snowball technique used for data collection which limits the generalizability of the findings. To overcome this limitation, our data were weighed by geographical location according to the list of CPs provided by the OPL. Thirdly, the collected data were also based on self-reported information which makes it prone to social desirability and might drive the results towards the null, leading to underestimation of some associations. Fourth, we were unable to assess the pandemic's impact on burnout due to a lack of data on pre-COVID burnout among CPs.

## Conclusion

An alarming prevalence of personal, work-related and client-related burnout was revealed among Lebanese CPs. The present study identified several modifiable factors that affect burnout among CPs in Lebanon. This study has many implications for practice and provides a framework for establishing policy interventions to reduce burnout levels among Lebanese CPs. Preventive strategies and interventions on individual and organizational basis like focusing on work–life balance, minimizing the level of chronic stress, increasing work satisfaction, peer support, counseling and self-care are recommended. Further studies exploring the independent and combined effect of the economic crisis on burnout levels among Lebanese CPs are highly recommended.

## Data Availability

The datasets generated during the current study are not publicly available, but are available from the corresponding author on reasonable request.
